# Adapting MOVED as a web-based moral elevation intervention for veterans with PTSD: Using feedback from a pilot trial and subject matter experts

**DOI:** 10.1016/j.conctc.2025.101445

**Published:** 2025-02-06

**Authors:** Adam P. McGuire, Alexander Riera, Xrystyan Lascano

**Affiliations:** a VISN 17 Center of Excellence for Research on Returning War Veterans, Waco, TX, USA; b Central Texas Veterans Health Care System, Temple, TX, USA; c Department of Psychology and Counseling, The University of Texas at Tyler, Tyler, TX, USA

**Keywords:** Treatment adaptation, Subject matter experts, PTSD, Veterans, Moral elevation, Positive psychology

## Abstract

**Background:**

Alternative, easily accessible treatment options are needed to aid efforts to address the negative effects of PTSD among veterans. One approach that has shown promise in a pilot trial is a moral elevation-based intervention titled, MOVED. Qualitative feedback from veterans in the pilot trial identified several strengths, but also highlighted opportunities to improve the intervention. In this adaptation phase, we incorporated feedback from pilot participants with input from subject matter experts (SMEs) to inform adaptation decisions using the Model for Adaptation Design and Impact (MADI) framework. In this paper, we outline the process and final adaptations decisions in preparation for a future efficacy trial to assess the impact of MOVED on targeted outcomes for veterans with PTSD.

**Method:**

We identified 10 SMEs that included veterans, clinicians, and researchers who participated in workgroup meetings to review 17 identified issues from the pilot and potential adaptations to address those concerns. We used the MADI framework to guide workgroup meeting discussions to determine what changes should be incorporated, including identifying potential negative outcomes for any adaptations and if they can be mitigated with other actions.

**Results:**

SMEs agreed with proposed adaptations for 15 of 17 issues and proposed mitigating measures for four of those adaptations to avoid anticipated negative outcomes. Two proposed solutions were refuted and not selected for adaptation.

**Conclusions:**

Using the MADI framework with input from SMEs allowed us to make informed decisions about adaptations for MOVED, thus contributing to further treatment development in preparation for a future efficacy trial.

## Highlights

• The MADI framework and expert input guided treatment adaptation decisions.
• 15 of 17 proposed changes were adopted with modifications to 4 solutions.
• This adaptation phase led to a refined version of MOVED for use in future trials.

## 1 Background

### 1.1 MOVED intervention

Veterans are at significant risk of trauma exposure and subsequently developing posttraumatic stress disorder (PTSD) [1,2]. There are several evidence-based practices (EBPs) that are available to treat PTSD symptoms, and past research has demonstrated strong support for the effectiveness of those interventions [3]; however, there are limitations to current approaches. There are known problems with treatment access for rural populations [4], barriers associated with stigma within military culture [5], and issues with engagement and retention [6]. Accordingly, veterans have expressed an interest in alternative treatment approaches [7]. Innovative paths to trauma recovery are needed to expand trauma care for veterans that includes viable alternative options. To address this need, we developed a novel, positive psychology intervention titled, MOVED: Moral Elevation Online Intervention for Veterans Experiencing Distress. MOVED is a web-based, self-guided intervention (i.e., no therapist) that features a unique focus on eliciting moral elevation—a positive emotional state of feeling uplifted and inspired after witnessing another person perform a virtuous act, which is linked with a range of relevant benefits including greater social engagement, self-improvement motives, and improved psychological health [8,9]. MOVED is well-suited to address known limitations because it offers an innovative positive psychology approach to trauma recovery as an alternative option, and it includes web-based and self-guided features that can increase accessibility and engagement with treatment.

In a pilot trial, we assessed the feasibility and acceptability of MOVED for post-9/11 veterans with probable PTSD and moral injury distress—strong guilt, shame, or anger about witnessing or perpetrating an act that was perceived as a moral transgression [10]. In this pilot, eligible veterans were randomized to either a treatment condition that included eight sessions of MOVED over four weeks, or a no-treatment control condition that asked veterans to complete eight sessions of online surveys over four weeks without any treatment content. Results indicated MOVED was feasible with successful enrollment and high levels of engagement in sessions [11]. Veterans also reported MOVED was acceptable, satisfactory, and linked with a wide range of psychosocial benefits as described in a posttreatment qualitative interview. Preliminary findings further supported potential benefits as indicated by large decreases in PTSD symptoms (*d* = 1.44), approximately twice that of veterans in the control condition (*d* = 0.71) and medium-sized increases in physical (*d* = 0.71) and psychological quality of life (*d* = 0.74) compared to no meaningful changes in the control condition. Notably, veterans in the treatment condition reported no changes in moral injury distress, whereas the control condition reported some improvement on total scores, but not on subscale scores of moral injury measurement. As noted in previous work, there are several potential explanations why this occurred, although it is ultimately unclear. These mixed findings, combined with qualitative feedback from veterans, suggest more work is needed regarding the assessment of moral injury in the context of this intervention, and that targeting moral injury may require a greater explicit focus on that issue. Therefore, we elected to advance to a refinement stage with a version of MOVED that focuses on PTSD symptoms exclusively, and to pursue alternative treatment options for moral injury in the future. Overall, results from the pilot trial provided strong, initial support for the potential benefits of MOVED with PTSD in veterans, warranting further investigation with a Stage 2 efficacy trial [12].

Prior to an efficacy trial, we aimed to optimize MOVED with adaptations that can further enhance engagement and boost effects for targeted outcomes. One source for identifying potential adaptations is qualitative feedback from the pilot. The pilot concluded with a 1-hour, semi-structured interview for all available veterans randomized to the treatment condition. Veterans were asked about a range of topics including initiation, engagement, session and treatment experiences, and any suggested revisions to MOVED. Interviews were transcribed, coded, and analyzed for emergent themes. Results from thematic analysis identified several areas for potential adaptation, which introduced multiple decision points about if or how to implement specific suggestions. To maximize the potential impact, we aimed to incorporate feedback from subject matter experts (SMEs) in the adaptation process. Our goal was to make adaptation decisions with input from three types of SMEs: 1) veterans with PTSD, 2) clinicians who provide trauma-focused treatment to veterans with PTSD in Veterans Health Administration (VHA) clinics, and 3) researchers with experience studying trauma-focused treatments for veterans that integrate technology. The purpose of this paper is to describe this adaptation phase and demonstrate the benefits of this approach. First, we will review the MOVED intervention and the components that were being considered for adaptation. Next, we will describe how SMEs were included to evaluate potential adaptations using the Model for Adaptation Design and Impact (MADI) framework [13]. Lastly, we will present key adaptation decisions based on input from SMEs and discuss the implications for using this adaptation approach.

### 1.2 MOVED intervention components

In the pilot trial, MOVED included eight sessions across four weeks. All sessions were accessed online and fully self-guided (i.e., no therapist). Participants received an email at 6:00am with a link to the assigned session every Monday and Thursday morning. A reminder email was sent to all participants who did not complete the session by 6:00pm. Participants who did not complete the session during the scheduled day received a phone call reminder the next morning and were encouraged to complete the session within the next 12 hours.

In Session 1, we presented psychoeducation materials including a definition of moral elevation and a description of several virtues that can trigger an elevation response (e.g., courage, selfless service). Across all sessions, MOVED included three key components: 1) elevation elicitation exercise, 2) reflection exercise, and 3) goal setting. The elevation elicitation exercise for Sessions 1–4 involved watching a brief video clip (2–4 min in length) of a moral exemplar demonstrating virtuous behavior. Elevation elicitation for Session 5–8 involved a recall exercise in which participants were asked to recall a time they witnessed virtuous behavior in their life.

For all sessions, participants completed a journal reflection exercise after the elicitation exercise. Participants used prompts to write (type) about their reactions to the elevation exercise and reflect on what they felt motived to do in response. Lastly, participants set a goal to complete before the next session. Goal setting involved prescribed goals for Sessions 1 and 2, and self-generated goals for Sessions 3–8. In the first two sessions, participants were asked to complete simple, social-oriented goals that involved telling another person about the elevation-eliciting videos. Starting with Session 3, they were asked to generate their own goal that met SMART criteria: a goal that is Specific, Measurable, Achievable, Relevant, and Timely. All subsequent sessions started with a brief check-in that asked participants to describe the outcome of their goal pursuit efforts.

### 1.3 Adaptation framework

The Model for Adaptation Design (MADI) framework is a guideline for implementing systemic adaptations to interventions that aims to consider the long-term impact of changes on the implementation process, the intervention itself, and targeted outcomes [13]. This is important because it is possible for new modifications to yield desired *and* undesired outcomes. Alternative approaches focus on describing adaptations with limited guidance or consideration of how proposed changes may impact various outcomes, which can lead to misunderstandings of results and limit the effectiveness of an intervention [14]. MADI facilitates proactive efforts towards future adaptations by facilitating an exploration of relationships between the constructs, intervention, and proposed changes to provide greater insight into potential impact.

MADI includes three main domains: adaptation characteristics (Domain 1), potential mediating and moderating effects (Domain 2), and implementation and intervention outcomes (Domain 3). Domain 1 focuses on what was adapted, for who, and defining the adaptation systematically. Domain 2 provides a set of considerations for the adaption, such as how well changes to an intervention fit within the intended context. Domain 3 focuses on the impact of the adaptation on the users of the intervention with consideration of critical factors like fidelity, adoption, and sustainability. The purpose of these domains is to help make changes to an intervention that are appropriate and acceptable without weakening its ability to have the desired effect. To fully respond to feedback from the MOVED pilot trial, we used the MADI framework while incorporating relevant SMEs including veterans, clinicians, and researchers. In this case, Domain 1 provided a context for identifying which adaptations should be considered, and Domain 2 guided the assessment of potential adaptations with input from SMEs. Domain 3 was not incorporated into the process described, but it will be considered during future trials when adaptations are implemented. Next, we will provide details about the SMEs who provided input, specific steps of adaptation decisions with the workgroups of SMEs, and final adaptation decisions.

## 2 Method

### 2.1 Subject matter experts

Ten SMEs were identified to form workgroups that evaluated proposed changes and provided feedback. SMEs were recruited from three categories of expertise on trauma-focused treatments: veterans, VHA-based clinicians, and researchers. All SME groups were recruited based on their unique experiences and exposure to what is considered feasible, satisfactory, and effective in treating PTSD symptoms among veterans. We wanted to seek additional insight from veteran SMEs with lived experience to help determine if proposed changes adequately addressed veterans’ suggestions from the pilot. We believed that clinicians providing care in VHA clinics could also offer an important perspective on how adaptations would align with strengths and weaknesses of existing treatments, as well as how they may impact implementation in a VHA setting. Lastly, we believed researchers with relevant expertise could offer additional insight into the feasibility of proposed changes in the context of pursuing a future efficacy trial and the scientific components of the treatment development process.

Veteran SMEs were recruited from a regional VHA PTSD clinic by asking for referrals of veterans who 1) have been diagnosed with PTSD, 2) completed at least one series of evidence-based practice treatment (e.g., Prolonged Exposure), and 3) self-reported as being familiar with veterans’ concerns regarding PTSD treatment. The lead psychologist of the regional PTSD clinic contacted a range of potential referrals that differed in age, gender, branch of service, and military history to inquire about their interest in participating as a SME. Six veterans consented to being contacted by the study team. Three veterans responded to further inquiries and agreed to participate in a workgroup meeting to provide feedback. All veteran SMEs self-identified as men with combat exposure serving in the Army (2) and Navy (1).

VHA-based clinicians were recruited from the Dissemination and Education Division of the National Center for PTSD. Division leadership provided a list of clinicians who were enrolled in the PTSD Mentoring Program with extensive training and experience administering trauma-focused treatments in a VHA setting. Six clinicians were contacted; four responded and consented to participate. Consenting clinicians were all licensed practitioners (LP or LISW-S) who differed in region of employment (East Coast, Midwest, South) and educational background (MSW, PsyD in Clinical Psychology, PhD in Clinical Psychology, PhD in Counseling Psychology).

Researchers were recruited based on prior experience with conducting clinical trials with veterans that also included technology integration (e.g., web-based sessions, virtual assessment, etc.). Three researchers were invited and agreed to participate, including two VA-based investigators and one investigator who has obtained funding from the Department of Defense to study veterans and servicemembers.

### 2.2 Procedure

***Pre-Workgroup Procedures***. First, initial feedback from veteran participants from the pilot was organized by the first author and arranged into seven broad domains for potential adaptation: 1) therapist, 2) goals, 3) videos, 4) recall exercises, 5) other session content, 6) intervention structure/schedule, and 7) other engagement issues. Across all domains, 17 potential issues were identified along with corresponding suggestions from veterans in the pilot (see Table 1). The first author generated an initial proposed solution for each issue based on the veterans’ suggestions from the pilot. A master document was created that included specific issues, suggestions, and proposed solutions across all domains. This master document was distributed to all SMEs for review before the workgroup meeting along with the full manual from the pilot, findings from the pilot, and supplemental materials related to specific potential changes.

**Table 1:** Overview of domains, issues, and suggestions generated from feedback in pilot trial.

Domain		Issue	Veteran Suggestion from Pilot (if present)	Proposed Solution	Outcome After Workgroup Meetings
Therapist	1	Wanted a therapist interaction to be included.	Add phone call with study therapist.	1 phone call (30–60 min) with therapist between Sessions 3–5.	Not adopted

Goals	2	Too difficult to generate session goals on their own.	Provide a set list of session goals tailored to each video.	Generate a new list of SMART goals; create separate list for each video.	Adopted with modifications
	3	Forgot content of session goals and forgot to do them in the first place.	Provide text reminders about goals.	Send texts halfway between goals to 1) remind veterans to complete goal, and 2) remind what goal was.	Adopted
	4	Too difficult to complete 2 goals with short turnaround time (2–3 days between sessions).	(none provided)	Simplify goals to increase feasibility of 2 goals per week.	Adopted with modifications
	5	Veterans seemed confused about goals to tell others about videos (Sessions 1 & 2).	(none provided)	Switch to using predetermined list of goals for all sessions.	Adopted
Videos	6	Some videos were “cheesy,” unrelatable, or elicited negative response.	Replace bad videos.	Review and update video set, including add more videos.	Adopted with modifications
	7	Wanted more videos.	Add more videos.	Increase the number of videos from 4 to 5 across 8 sessions.	Adopted
	8	Wanted access to new videos after treatment.	Add supplemental videos for booster sessions.	Identify 2–4 new videos and list of goals for each video that can be used for booster sessions after Session 8 (i.e., resource made available to veterans).	Adopted
Recall Exercises	9	Difficulty being able to recall witnessing a virtuous behavior in daily life.	(none provided)	Clarify instructions: Encourage veterans to 1) recall any virtuous behavior they may have witnessed at any point in their life (i.e., not just in the past few days), or 2) identify virtuous behavior from online sources (e.g., social media, news outlets, etc.).	Adopted with modifications

Other Session Content	10	Did not directly address trauma.	Add more trauma-focused content.	Include new videos that feature trauma and recovery more explicitly.	Adopted
	11	Could not access or review previous sessions (and content).	Provide access to old sessions.	Create link that includes all material for previous sessions (i.e., shared drive).	Adopted
	12	Session 1 could be clearer.	(none provided)	Update description of moral elevation in Session 1 (both cartoon and text).	Adopted
	13	Some content could be better tailored to veterans.	Use military terms throughout the intervention.	Incorporate a list of changes with proposed military terms/language.	Not adopted
Intervention Structure/Schedule	14	Felt less engaged during Sessions 5–8 with recall exercises and wanted more videos.	Change schedule to increase number of videos presented and alternate between video/recall.	New format (5 videos, 3 recall exercises): S1-S3 Video 1–3; S4 Recall Exercise 1; S5 Video 4; S6 Recall 2; S7 Video 5; S8 Recall 3.	Adopted
	15	Not enough time between sessions that asked for a recall of witnessing virtuous behavior.	Change format so recall exercises are not back-to-back.	Same solution for #14.	Adopted
	16	When checking-in at the beginning of session, it was difficult to remember last session's goal/objective.	Provide more detailed information about previous session during initial check-in.	Include tailored content that presents veteran's specific goal and other relevant info from previous week during check-in.	Adopted
Other Engagement	17	Difficulties remembering sessions and tasks.	Provide option to choose text or email reminders.	Allow the option to opt-in for text or email reminders about: 1) upcoming sessions, and/or 2) designated goal and deadline.	Adopted

*Note*. S = Session.

***Workgroup Meetings***. Next, two virtual workgroup meetings were scheduled to discuss potential adaptations. The first meeting was scheduled with researchers and clinicians. A separate meeting was scheduled with just veteran SMEs to ensure they felt comfortable speaking openly about their opinions and perspectives. In both meetings, the first author facilitated a discussion for each proposed solution by using the MADI framework, which informed discussions about whether an adaptation should be implemented. Meetings focused on three key questions from the MADI framework: 1) *is the adaptation systemic, designed with a goal in mind, and aligned with the core functions of the intervention?*; 2) *are there any predicted negative impacts on outcomes (intended or unintended)?*; and if so, 3) *can we mitigate negative impacts with implementation strategies or offset with positive impacts on other outcomes?* Each meeting involved discussions between SMEs for potential adaptations until consensus agreement was reached for a recommendation. Specifically, the questions above were posed to each group regarding potential changes, and the ensuing discussion within each meeting involved probing for responses, identifying agreements or disagreements, and facilitating discussion about any necessary adjustments to proposed solutions until consensus was reached. The first and second authors combined feedback from both meetings, then used that information to identify viable adaptations for all issues raised. If workgroup meetings were unable to reach consensus after discussion, or if there was a lack of consensus between veteran and clinician/researcher workgroup meetings, the first author (intervention developer) would make a final determination based on the arguments provided.

## 3 Results

Following the workgroup meetings, SMEs agreed with proposed solutions for 15 of the 17 issues with consensus across clinician-researcher and veteran meetings (see Table 1). Among the 15 agreed-upon solutions, SMEs anticipated potential negative outcomes with four of those solutions, and accordingly, proposed mitigating measures to avoid anticipated negative outcomes. Notably, there were no disagreements on potential solutions between the two, independent workgroup meetings.

### 3.1 Proposed adaptations: Adopted

#### 3.1.1 Goals

The first two changes that were adopted without modification pertained to the goal setting activity of MOVED. Veterans from the pilot noted they sometimes forgot their session goal and forgot to pursue that goal in the days between sessions. Several veterans endorsed a suggestion to provide text reminders about the specific details of the goal and the deadline for completing that goal. We proposed to send a text at the halfway point between sessions for any veteran who consented to receiving text reminders, which was approved by both workgroups. Additionally, some veterans expressed confusion about the predetermined goals during Sessions 1 and 2, which asked veterans to discuss the contents of the elevation video with another person. We proposed removing those assigned goals and switching to a predetermined *list* of goals for each session that veterans could choose from, which would allow veterans to select a specific goal that might be most relevant or helpful to them. See “*Provide List of Goals to Select*” in the adopted with modifications section below for more details about changes to the goal selection process.

#### 3.1.2 Videos

On average, most veterans described enjoying the videos. Many offered a suggestion to add more videos and to create opportunities for watching videos after Session 8 as follow-up or booster sessions. In response, we proposed to increase the number of videos shown from four out of eight sessions in the pilot to five out of eight, which was approved by both workgroups. This allowed for more exposure to videos while still retaining the recall exercise for the remaining three sessions, which is designed to help veterans learn how to seek out elevation-eliciting stimuli on their own. We also proposed to add 2–4 optional booster sessions. Each session would include a new elevation video, and all boosters would be available to all veterans after Session 8. This solution was endorsed by both workgroups.

#### 3.1.3 Other session content

Veterans identified other issues and suggestions with the session content including 1) add more trauma-focused content, 2) provide access to completed sessions, and 3) clarify the psychoeducation content about elevation in Session 1. Both groups agreed to the proposed solutions of enhancing clarity of Session 1 by improving the readability of the text and updating the supporting graphics or visuals. Both groups also agreed to the solution of providing access to old sessions by sharing links to a publicly accessible website or platform that would be available for veterans enrolled in MOVED. Lastly, we proposed to add new elevation videos that featured survivors of trauma and that highlighted their recovery. This was discussed as an opportunity to incorporate trauma content without shifting too far to include explicit process work of traumatic events that is involved in traditional psychotherapy, which often requires working with a therapist for proper guidance and support. This solution was also agreed upon by both groups.

#### 3.1.4 Intervention structure/schedule

Regarding the intervention schedule, several veterans identified issues with feeling less engaged during the final four sessions in which they are asked to complete a recall exercise (instead of watching a video), and that it was difficult to identify elevation-eliciting stimuli in daily life for four sessions in a row. We proposed a new format that would incorporate more videos (consistent with other suggestions; see above) and alternate between sessions that include recall versus video elevation exercises, starting with Session 4. Alternating the elevation exercise type would also result in one video and one recall exercise per week starting in week 2, which could increase the feasibility of completing the recall exercises. Lastly, veterans noted checking-in at the beginning of each session and answering questions was difficult if they could not remember what occurred in the previous session. We proposed to include tailored content at each check-in that provided a review of what was covered in the previous session and what goal was selected for each veteran. All three solutions were agreed upon by both workgroups.

#### 3.1.5 Other engagement

Related to previous concerns about difficulties remembering their assigned session goal, some veterans described difficulties remembering to start and complete sessions. In the pilot, the primary method of reminding participants to start a given session was email; therefore, some veterans suggested there should be an option to receive text reminders instead of email. In addition to providing text reminders about completing goals between sessions (see “*Goals*” section above), we proposed to offer each veteran the option to choose whether they wanted text or email reminders to log on or start each session, which was agreed upon by both groups.

### 3.2 Proposed adaptations: Adopted with modifications to mitigate potential negative outcomes

#### 3.2.1 Provide list of goals to select

Several veterans from the pilot indicated it was difficult to create goals after each session and many suggested that having a list of goals to choose from would boost goal attainment. Clinicians and researchers identified a potential negative outcome to this solution; namely, there is a risk that veterans could be less inclined to pursue a goal selected from a predetermined list if the goal is disconnected from their personal values or if they do not perceive a sense of “ownership” over the goal. To mitigate this outcome, clinicians advised incorporating minor modifications that could allow for greater personalization of each goal. One suggestion was to ask a follow-up question that prompts veterans to personalize an aspect of that goal (e.g., *Call a friend or family member to offer* support → *Who could you call, specifically?*). Another suggestion was to incorporate a values assessment in Session 1 and link values to each goal to encourage greater personal alignment in the goal selection process. Both strategies were deemed feasible and added to the proposed solution.

#### 3.2.2 Keep two simple goals per week

Veterans from the pilot noted difficulties with completing two goals each week (one per session). A proposed solution was to simplify the goals to increase feasibility of attainment while maintaining two goals per week. Clinician and researchers noted that if veterans were still unable to complete multiple goals per week, they could become discouraged and less engaged—potentially influencing treatment outcomes. The proposed solution to mitigate this risk was to provide more information at the beginning of the intervention that clearly outlines expectations. Veterans could be encouraged to set attainable goals (consistent with the SMART goal setting format) and perhaps easier goals at the beginning. This could be supported by the last solution of providing a list of attainable goals to choose from, instead of relying on veterans to generate their own goals.

#### 3.2.3 Replace ineffective videos

Although most veterans from the pilot described elevation-eliciting videos as a strength of MOVED, for those who did not have a positive reaction, they suggested replacing videos that were cheesy or unrelatable to veterans. We showed several videos, some from the pilot and some new videos for consideration, to the veteran SMEs to obtain direct feedback about this concern. Veterans confirmed that videos of veterans engaged in social connection were impactful; however, they noted a potential negative outcome for videos that appeared too specific. For example, we presented a new video clip about a veteran who demonstrated courage and perseverance by disclosing he was a survivor of military sexual trauma (MST), who described how he overcame distress during his recovery journey. Veteran SMEs noted videos/stories that featured specific traumatic experiences, especially MST, could lead to the same concerns about unrelatability. They noted that some veterans may be prone to see differences with the veteran in the video as an indication the intervention is not a good fit for them. In response, we decided to offer the video about MST in an optional booster session (a part of Adoption #8; see Table 1) instead of including it in the standard eight sessions, so that veterans can seek out that specific video content, if desired. We also reviewed all other videos to ensure broad applicability to veterans with PTSD.

#### 3.2.4 Clarify instructions for recall exercise

Some veterans reported difficulties with engaging in the recall elevation exercise that asked them to remember a moment when they witnessed virtuous behavior in daily life (compared to watching a video clip). We proposed a solution of expanding the recall exercise by encouraging veterans to recall any virtuous event from their life and to search for virtuous behavior using online sources (e.g., social media, positive news), if needed. However, clinicians and researchers noted simply expanding the potential source of elevation without more guidance could still result in difficulties connecting with the emotional experience. They also noted that perceived dependence on memory could be problematic given the potential for memory impairment with trauma exposure. To offset these potential issues, SMEs suggested providing more guidance by presenting veterans with specific examples of virtuous behavior they might have witnessed in the past, as well as fictional examples from movies or TV shows that could be easily accessed. Additionally, SMEs encouraged the integration of other strategies to increase the salience of this exercise, including guided instructions to mindfully visualize the virtuous behavior and prompts to recall physiological reactions to witnessing such behaviors. These modifications were deemed feasible and added to the list of adaptations.

### 3.3 Proposed adaptations: Not adopted

#### 3.3.1 Talking to a therapist

The first refuted adaptation was the proposal to add one 30- to 60-min phone call with a therapist at the midway point of the intervention. This was proposed in response to several veterans from the pilot who expressed an interest in speaking with a therapist during the intervention. Clinicians and researchers reported concerns that this addition could lead to several negative outcomes including 1) some veterans not feeling comfortable enough to open-up to a therapist in such a short period of time, 2) some veterans sharing too much and not getting a sufficient amount of follow-up or continuation of care if needed, 3) implementation problems with how to document such phone calls in clinics and accounting for the expected time commitment from future clinicians, and 4) concerns this requirement would significantly limit the scalability of MOVED (an otherwise self-guided intervention).

Veteran SMEs also expressed concerns that having a planned phone call may lead some veterans to feel “forced” to talk with a therapist. They noted many veterans are not ready to speak with a therapist, which may lead to significant resistance to the whole intervention. One veteran described his concerns by saying, “*I'll tell you from personal experience, I [have been] forced to talk to a therapist and I just didn't like it. [That felt] like an infringement, you know?*” Instead, all SMEs agreed it would be helpful to incorporate an option to request a referral if the veteran wanted more traditional treatment in addition to this self-guided intervention. In response, we decided to add a referral seeking feature, but will not incorporate a therapist phone call as a core component of MOVED.

#### 3.3.2 Incorporate military terms/language

The second refuted adaptation was the suggestion to add more military terms throughout MOVED. This was also proposed by veterans from the pilot who posited that veterans might be more successful at engaging with and completing intervention activities if they were aligned with known terms and schemas from their military training. The proposed solution included adding terms such as *mission* (referring to the overall intervention), *after action review* (check-in), *operation assessment* (journal response), *objective* (behavioral goal), and several others. Clinicians and researchers expressed concerns that such language could lead to negative reactions for veterans who had negative experiences in the military. They were also concerned that using this language could evoke characteristics of the military that are not necessarily conducive to successful individual therapy such as an emphasis on doing what is best for the collective (i.e., not prioritizing the individual) as well as the overuse of language that could be perceived as cold or emotion-less.

Veteran SMEs also noted the risk that military language could trigger a negative reaction for some veterans. One veteran stated, “*Don't try to bring the military side into it, because for some of us, that's what we're fighting against.*” They also noted other concerns about the implementation of this change—highlighting that several military terms differ between branches; therefore, using a term that applies to certain branches but not others could lead some veterans to feel alienated, forgotten, or misunderstood as members of another branch. They also described significant concerns that veterans might perceive the intervention as being disingenuous if veterans can easily identify language generated from a civilian with no real-life experience in the military. One veteran stated, “*You can tell the ones that aren't real. The best advice I could give you is just be genuine. Don't try to get to our level in that way*.” In response, we decided to not incorporate any new military terms in the adapted version.

## 4 Discussion

Quantitative results from the MOVED pilot trial provided strong support for initial feasibility, acceptability, and potential impact on quality of life and PTSD symptoms. Qualitative findings corroborated these results, but also indicated several areas of potential improvement to MOVED; however, it was unclear which suggestions should be implemented. Therefore, we elicited input from SMEs using the MADI framework to guide adaptation decisions. This process resulted in the confirmation of 11 adaptations as presented, four modifications of adaptations to prevent potential negative outcomes, and two rejected adaptations. This approach aided the decision process and resulted in several important benefits that support our ongoing efforts for successful treatment development.

One clear benefit to this approach was the ability to incorporate feedback from three SME types, all of whom have unique perspectives on PTSD treatment for veterans. Researchers provided input with the context of how specific suggestions might fit with previous empirical evidence and with consideration of future clinical trials. Clinicians provided insight into the direct implementation of these changes and the intervention—helping bridge the gap between designing an intervention for a research trial and designing an intervention for sustained use in the field. Veterans provided critical insight about the potential experiences of veterans, which was vital given our goal to make MOVED more accommodating for this population, specifically. This range of perspectives proved to be helpful as indicated by several points of feedback by each group regarding modifications. It was also helpful when there was agreement across all SMEs, which increased confidence in adaptation decisions.

Beyond multiple perspectives, the MADI framework proved to be a useful strategy in mitigating potential negative outcomes of any adaptation. Previously, we were primarily focused on the feasibility of any potential changes and their coherence with the overall treatment. However, the questions within Domain 2 of MADI elicited a series of conceivable negative outcomes that could arise from well-intentioned adaptations—resulting in four concrete modifications generated by the SMEs to reduce the likelihood of negative outcomes.

Lastly, the decision to abandon two major adaptations that could have fundamentally altered the intervention highlights the benefit of this method. SMEs were able to account for the bigger picture of how MOVED can best serve veterans with PTSD while considering their own experiences in this area to advise against directly incorporating a therapist into the intervention. Similarly, SMEs were able to identify that altering intervention language to more closely align with military language could have inadvertently led to negative outcomes and reduced engagement. SME perspectives combined with the MADI framework helped inform these important decisions.

### 4.1 Limitations

There are some limitations to this adaptation method that should be considered. First, SMEs were limited to 10 people, which may not fully represent all the experiences of the targeted population and settings. Notably, veteran SMEs were limited to three males, despite our efforts to recruit a diverse set of SMEs with respect to military and demographic characteristics, including gender. Although future research should incorporate a larger and more diverse set of veteran SMEs, few treatment-development studies document or incorporate veterans’ perspectives on both initial pilot feedback and refinement stages—indicating a strength of this study. Next, the initial feedback from pilot was not separated and analyzed by important demographic categories such as race, age, and gender; therefore, the adaptations to MOVED did not involve explicit discussions about the impact across these categories. Future trials of MOVED should be mindful of potential differences across important demographic characteristics and probe for how these adaptations could disproportionally impact one group over another, as needed. Future adaptation efforts should also consider ways to incorporate these considerations directly into the refinement process. Third, Domain 2 of the MADI framework provides a helpful guide to anticipating negative outcomes for each adaptation; however, these anticipated consequences are not rooted in any observable data from the intervention until adaptations are directly tested. Yet, the strength of this guided approach is that it could increase the likelihood of pre-emptively circumventing potential issues in a quick and cost-effective manner. The core limitation is that other issues could arise that were not considered, or the expected negative outcomes may not have occurred in the first place. Consistent with Domain 3 of the framework, future trials of MOVED should aim to monitor anticipated and unanticipated negative outcomes that could be attributed to adapted changes from the original pilot.

### 4.2 Conclusion

Despite limitations, this approach combined with the insight of key SMEs allowed us to make informed decisions about adaptations for MOVED. As we pursue a larger efficacy trial, we aim to conduct ongoing assessments of the potential impact of adaptations by measuring feasibility and engagement at session- and overall intervention levels. Beyond the efficacy trial, additional work is also required to transition this intervention and its components from being delivered through a research study to a sustainable tool that can be accessed by veterans and clinicians, independent of a research team. Through this ongoing process, we hope to advance the scientific understanding of the intervention's effects, as well as implement important changes that will expand engagement and benefits for veterans with PTSD.

## CRediT authorship contribution statement

**Adam P. McGuire:** Writing – review & editing, Writing – original draft, Project administration, Investigation, Formal analysis, Data curation, Conceptualization. **Alexander Riera:** Writing – review & editing, Writing – original draft, Project administration, Data curation. **Xrystyan Lascano:** Writing – review & editing, Writing – original draft, Resources.

## Declaration of competing interest

The authors declare that they have no known competing financial interests or personal relationships that could have appeared to influence the work reported in this paper.

## Data Availability

No data was used for the research described in the article.
